# Lateralising reverse shoulder arthroplasty using bony increased offset (BIO-RSA) or increasing glenoid component diameter: comparison of clinical, radiographic and patient reported outcomes in a matched cohort

**DOI:** 10.1186/s10195-024-00764-4

**Published:** 2024-04-18

**Authors:** Arno A. Macken, Geert Alexander Buijze, Michael Kimmeyer, Tilman Hees, Denise Eygendaal, Michel van den Bekerom, Laurent Lafosse, Thibault Lafosse

**Affiliations:** 1 Alps Surgery Institute, Clinique Générale d’Annecy, 4 Chem. de La Tour La Reine, 74000 Annecy, France; 2 Department of Orthopaedic Surgery and Sports Medicine, Erasmus Medical Centre, Dr. Molewaterplein 40, Rotterdam, the Netherlands; 3 Department of Orthopedic Surgery, Amsterdam UMC, Meibergdreef 9, Amsterdam, the Netherlands; 4 Department of Orthopedic Surgery, Montpellier University Medical Center, Lapeyronie Hospital, University of Montpellier, 371 Av. du Doyen Gaston Giraud, Montpellier, France; 5 OLVG Hospital, Jan Tooropstraat 164, Amsterdam, the Netherlands; 6 VU University Amsterdam, De Boelelaan 1105, Amsterdam, the Netherlands

**Keywords:** Bony increased offset reverse shoulder arthroplasty, Patient-reported outcomes, Range of motion

## Abstract

**Background:**

This study aims to compare the range of motion (ROM) of reverse shoulder arthroplasty lateralised by bony increased offset (BIO-RSA) using a standard 38-mm (mm) component to regular reverse shoulder arthroplasty (RSA) lateralised by using a 42-mm glenoid component. The secondary aims are to compare patient-reported and radiographic outcomes between the two groups.

**Materials and Methods:**

All patients with a BIO-RSA and size 38 glenosphere were retrospectively identified and matched to patients with a regular RSA and size 42 glenosphere. Matched patients were invited for a follow-up visit. ROM was assessed as well as radiographic outcomes (lateralisation, distalisation, inferior overhang, scapular notching, heterotopic bone formation, radiolucency, stress shielding, bone graft healing and viability and complications) and patient-reported outcomes (subjective shoulder value, Constant score, American Shoulder and Elbow Surgeons, activities of daily living which require internal rotation, activities of daily living which require external rotation and a visual analogue scale for pain). Outcomes were compared between the two groups.

**Results:**

In total, 38 BIO-RSAs with a size 38 glenosphere were matched to 38 regular RSAs with a size 42 glenosphere. Of the 76 matched patients, 74 could be contacted and 70 (95%) were included. At the final follow-up, there were no differences between the two groups in ROM, patient-reported outcomes or radiographic outcomes (*p* > 0.485).

**Conclusions:**

Using a larger glenosphere is a feasible alternative to BIO-RSA for lateralising RSA, providing comparable ROM, patient-reported and radiographic results, while potentially decreasing costs, operative time and complication rates.

*Level of evidence* III.

**Supplementary Information:**

The online version contains supplementary material available at 10.1186/s10195-024-00764-4.

## Introduction

The introduction of the reverse shoulder arthroplasty (RSA) design by Grammont revolutionised surgical treatment for shoulder pathologies [1]. However, it came with several drawbacks including prosthetic instability, deficient internal and external rotation, aesthetic complaints owing to loss of shoulder contour, scapular impingement and stress fractures [2]. All of these can be attributed completely or partially to the medialisation and distalisation of the humerus and the centre of rotation.

One option to lateralise the glenoid component is bony increased offset reverse shoulder arthroplasty (BIO-RSA) [3]. Some studies report improved rotation with BIO-RSA compared to non-lateralised RSA [4, 5]. However, this procedure is promising but also more technically challenging, prone to specific compilations and costly compared with regular RSA [6].

Increasing the size of the glenoid component has also been proposed to further reduce the rate of scapular notching and improve rotational range of motion (ROM) by lateralising the humerus without changing the centre of rotation, and by increasing the inferior overhang. Previous studies have reported lower rates of scapular notching and greater rotational and elevation ROM in patients with a larger glenoid component [4–6]. However, other studies did not replicate these results [5, 7].

To our knowledge, no prior studies have been published directly comparing these two groups. To address the gaps and contradictions in the literature, this study aims to compare the ROM of BIO-RSA using a 38-mm (mm) component with regular RSA using a 42-mm glenoid component in a matched retrospective series using the Delta Xtend reverse shoulder prosthesis (DePuy Synthes, Warsaw, USA) with a 155 ° neck-shaft angle design. The secondary aims are to compare patient-reported and radiographic outcomes, such as scapular notching.

## Methods

### Patient selection

After approval from the institutional review board, all consecutive primary RSA procedures performed between January 2015 and December 2021 were identified. Because all consecutive patients were identified, no power calculation was performed. Inclusion and exclusion criteria are reported in Table 1.

**Table 1:** Inclusion and exclusion criteria

Inclusion	Exclusion
1. RSA using Delta Xtend* model	1. Deceased patients
2A. Regular RSA + size 42 glenosphere or 2B. BIO-RSA + size 38 glenosphere	2. Language barrier with regards to the researchers (speaking English, French, Italian, German, Dutch and Spanish)
	3. No contact information
	4. Bone graft used for glenoid bone loss or glenoid defects (instead of lateralisation)
	5. Augmented or lateralised prosthesis designs
	6. Preoperative nerve palsies or neurological defects

*BIO*-*RSA* bony increased offset reverse shoulder arthroplasty, *RSA* reverse shoulder arthroplasty

*Depuy Synthes, Warsaw, USA

All patients with a BIO-RSA and a size 38 glenosphere (BIO-RSA 38 group) were matched with patients with a regular RSA and a size 42 glenosphere (RSA 42 group) with a 1:1 ratio. Patients were matched based on sex, age, body mass index (BMI) and the indication for RSA using optimal pair matching. The mean and maximum distances in propensity score between the pairs were reported. The matched patients were contacted for a follow-up visit. In cases where patients were unable to visit the hospital, questionnaires were completed via telephone. The minimum follow-up for inclusion was set at 1 year, on the basis of a previous study that reported no change in ROM and patient reported outcome measures (PROMs) between the 1- and 2-year follow-up periods [8].

### Variables

A revision was defined as any unplanned surgical procedure to the ipsilateral glenohumeral joint related to the arthroplasty. A complication was defined as any unforeseen medical problem caused by the RSA procedure which negatively influences the outcome temporarily or permanently [9].

The following questionnaires were completed: subjective shoulder value (SSV) [10], Constant score [11], American Shoulder and Elbow Surgeons (ASES) [12], activities of daily living which require internal rotation (ADLIR) [13, 14], activities of daily living which require external rotation (ADLER) [15] and a visual analogue scale (VAS) for pain.

### Radiographic outcomes

On the most recent radiographic imaging lateralisation, distalisation, inferior overhang, scapular notching, heterotopic bone formation, radiolucency, stress shielding, bone graft healing and viability and potential other complications were independently assessed by two authors in a standardised fashion described in Additional file 1: Table S1 [3, 16–24]. All assessments were then discussed with the senior author to reach a consensus between the three assessors. For the angle and distance measurements, three authors including the senior author independently performed the measurements, and the mean result was calculated.

### Statistics

The improvement from pre- to postoperative measurements was compared using paired Wilcoxon signed-rank tests. For the comparisons between the two groups (BIO-RSA 38 versus RSA 42), unpaired tests were used. This was chosen over paired tests owing to the potential differences in response rate between the groups leading to unequal group sizes, the overall small cohort and limited population to draw from for patient matching leading to minimal dependence between matched cases [25, 26]. Chi-squared or Fisher exact tests were used for binary categorical variables and *T*-tests or Mann–Whitney *U* tests were used for continues variables.

For the radiological assessment, reliability between the first two authors analysing the radiographs was assessed using the interclass correlation (ICC) for the angle measurements and Cohen’s kappa (*k*) for the grades. An ICC of less than 0.50 was considered poor reliability, between 0.5 and 0.75 moderate reliability, between 0.75 and 0.9 good reliability and greater than 0.9 was considered excellent reliability. A Cohen’s kappa of less than 0.20 was considered a slight agreement, between 0.21 and 0.40 fair, between 0.41 and 0.60 moderate, between 0.61 and 0.80 substantial and between 0.81 and 1.00 was considered almost perfect agreement [27].

To correct for multiple testing, *p*-values were adjusted using a Benjamini–Hochberg procedure. An adjusted *p*-value lower than 0.05 was considered statistically significant. A post-hoc power calculation was performed for the primary outcomes (rotational range of motion) using 0.05 as the significance level, a resulting power of > 0.80 was considered sufficient. Statistical analysis was performed using R version 4.2.1 (R Foundation for Statistical Computing, Vienna, Austria).

### Surgical technique

In all cases a Delta Xtend prosthesis was used (DePuy Synthes, Raynham, USA) with a high-mobility polyethylene insert size 3. A deltopectoral approach was used for all BIO-RSA cases and an anterosuperior approach for all RSA cases. For BIO-RSA cases, a bone graft of approximately 1 cm in width was used, harvested from the resected humeral head when possible. In cases of BIO-RSA the glenoid baseplate construct was angled 10 ° inferior, in RSA cases an inclination angle of 0 ° was aimed for. The subscapularis tendon was either absent or detached in all cases without subsequent repair.

## Results

**Fig. 1:**
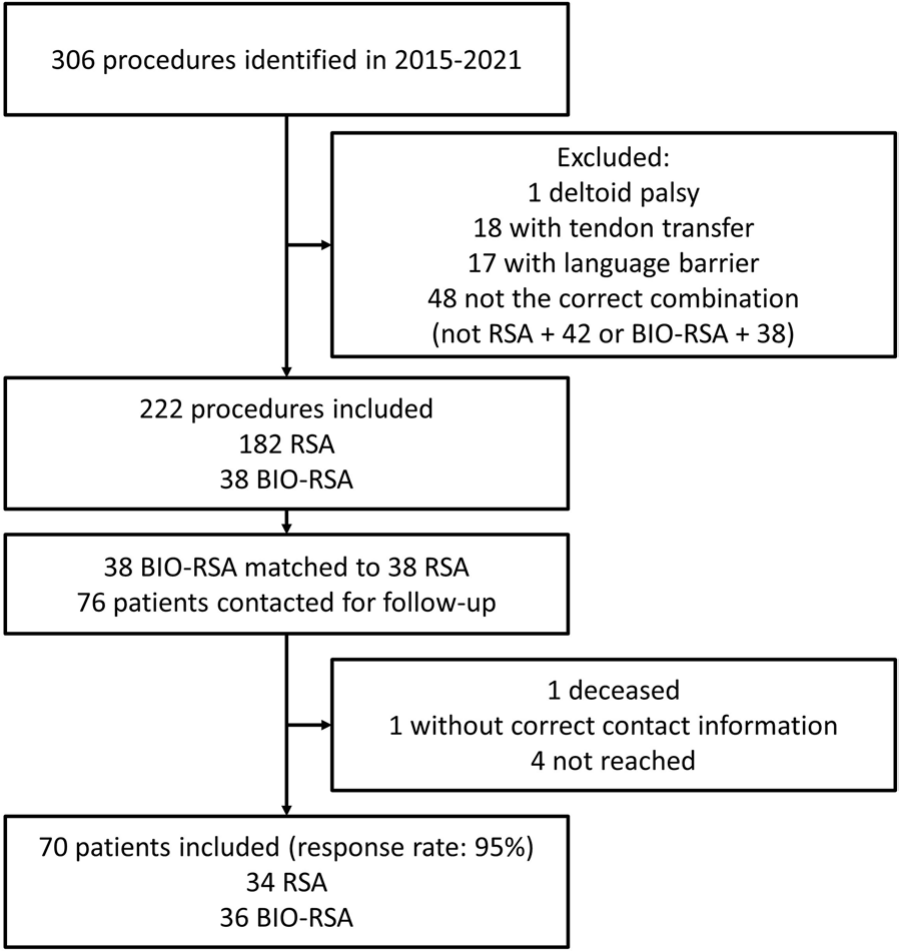
Inclusion flowchart

After inclusion (Fig. 1) 38 BIO-RSAs with a glenosphere size 38 were matched to 38 regular RSAs with a glenosphere size 42. The median distance in propensity scores between the matched pairs was 0.27 and the maximum distance was 0.58. Of the 76 matched patients, 74 could be contacted and 70 were included (response rate: 95%). In total, five patients had a bilateral prosthesis but both shoulders were not included in any cases. The post-hoc power calculation resulted in a statistical power of > 0.99 for the primary outcomes (rotational range of motion).

### Study cohort

The mean age at the time of primary surgery in the cohort was 72 (*SD* 8) years and the majority of patients were female (44/70, 63%). The follow-up was longer in the RSA 42 group [3.7 years; interquartile range (IQR): 2.2–5.4 versus 2.3 years; *IQR* 2.1–2.5, *p* = 0.0126). The other patient characteristics did not differ between the groups after correction of the *p*-values (Table 2).

**Table 2:** Patient characteristics

	BIO-RSA 38 (*n* = 36)	RSA 42 (*n* = 34)	*p*-value	Adjusted *p*-value
Female, *n* (%)	22 (61)	22 (65)	^A^0.756	1.000
Age, mean years (SD)	70 (8)	73 (7)	^**B**^**0.0424**	0.806
BMI, mean kg/m^2^ (SD)	26 (4)	26 (4)	^B^0.990	1.000
Diagnosis, *n* (%)			^C^0.912	1.000
Osteoarthritis	13 (36)	14 (41)		
Cuff tear arthropathy	12 (33)	9 (26)		
Irreparable cuff tear	10 (28)	10 (29)		
Acute fracture	1 (3)	1 (3)		
ASA classification, *n* (%)			^A^0.938	1.000
I	8 (24)	15 (42)		
II	21 (62)	15 (42)		
III	5 (15)	6 (17)		
Comorbidities, *n* (%)				
Diabetes	3 (8)	2 (6)	^C^1.000	1.000
Cardiological	20 (56)	18 (53)	^A^0.826	1.000
Thyroid disease	5 (14)	4 (12)	^C^1.000	1.000
Gastroenterological	4 (11)	4 (12)	^C^1.000	1.000
Respiratory	0 (0)	3 (9)	^C^0.109	1.000
Urological	4 (11)	3 (9)	^C^1.000	1.000
Neurological	1 (3)	5 (14)	^C^0.199	1.000
Psychological	2 (6)	1 (3)	^C^0.609	1.000
Oncological	0 (0)	1 (3)	^C^0.486	1.000
Smoking, *n* (%)	5 (14)	3 (9)	^C^0.711	1.000
Dominant side operated, *n* (%)	12 (52)	7 (32)	^A^0.167	1.000
Previous surgery, *n* (%)	12 (33)	6 (18)	^A^0.133	1.000
Rotator cuff	11 (31)	3 (9)	^**A**^**0.0231**	0.462
Latarjet	1 (3)	2 (6)	^C^0.609	1.000
Other	1 (3)	1 (3)	^C^1.000	1.000
Follow-up time, median years (IQR)	2.3 (2.1–2.5)	3.7 (2.2–5.4)	^**D**^**0.0006**	**0.0126**

*BMI* body mass index, *BIO*-*RSA* bony increased offset reverse shoulder arthroplasty, *RSA* reverse shoulder arthroplasty, *SD* standard deviation

^A^chi-square

^B^*t*-test

^C^Fisher exact test

^D^Mann–Whitney *U* test

Acromioplasty was more commonly performed in the RSA 42 group (32/34, 94% versus 25/36, 69%, *p* = 0.0399). The other treatment characteristics did not differ between the two groups (Table 3).

**Table 3:** Treatment characteristics

	BIO-RSA 38 (*n* = 36)	RSA 42 (*n*= 34)	*p*-value	Adjusted *p*-value
Acromioplasty, *n* (%)	25 (69)	32 (94)	^**A**^**0.00798**	**0.0399**
Humerus size, median (IQR)	10 (10–11)	10 (10–11)	^D^0.707	0.707
Cemented humerus, *n* (%)	0 (0)	3 (9)	^C^0.109	0.327
Retroversion, median ° (IQR)	30 (30–30)	30 (30–30)	^D^0.588	0.707
Locking screws, *n* (%)			^A^0.069	0.276
2/4	0 (0)	17 (50)		
0/4	36 (100)	17 (50)		
Graft donor, *n* (%)				
Humeral head	34 (94)			
Iliac crest	1 (3)			
Allograft	1 (3)			

*BIO*-*RSA* bony increased offset reverse shoulder arthroplasty, *IQR* interquartile range, *RSA* reverse shoulder arthroplasty

^A^chi-square

^B^*t*-test

^C^Fisher exact test

^D^Mann–Whitney *U* test

Information on preoperative assessments was available in 67 patients (96%). There was no difference between the groups in preoperative PROMs and ROM (*p* > 0.260; Table 4).

**Table 4:** Preoperative measurements

Median (IQR)	BIO-RSA 38 (*n* = 36)	RSA 42 (*n* = 34)	*p*-value	Adjusted *p*-value
Subjective Shoulder Value (0–100)	30 (30–50)	40 (30–48)	0.554	0.554
VAS pain (0–10)	6 (5–7)	7 (5–7)	0.348	0.554
Anterior elevation, °	90 (70–130)	105 (80–137)	0.547	0.554
External rotation, °	10 (−4 to 30)	20 (10–44)	0.065	0.260
Internal rotation, level reached	buttock (hip-L3)	L3 (buttock-T12)	0.059	0.260

*BIO*-*RSA* bony increased offset reverse shoulder arthroplasty, *IQR* interquartile range, *RSA* reverse shoulder arthroplasty, *VAS* visual analogue scale

### Patient-reported outcomes

PROM results at final follow-up were available in 67 patients (96%). The SSV and pain score at final follow-up improved significantly compared with the preoperative measurements (*p* < 0.001), the other PROMs were not recorded preoperatively. There were no differences between the two groups in PROMs at the final follow-up or the amount of improvement between preoperative measurements and the final follow-up (*p* = 0.961, Table 5).

**Table 5:** Patient-reported and clinical outcomes

	BIO-RSA 38 (*n* = 36)	RSA 42 (*n* = 34)	*p*-value	Adjusted *p*-value
At final follow-up				
Subjective shoulder value (0–100), median (IQR)	80 (70–91)	80 (60–90)	^D^0.488	0.961
VAS pain (0–10)), median (IQR)	1 (0–2)	1 (0–3)	^D^0.615	0.961
Constant score, mean (SD)	62 (17)	65 (23)	^B^0.699	0.961
ASES score, median (IQR)	82 (75–90)	82 (67–92)	^D^0.790	0.961
ADLIR score, median (IQR)	84 (78–88)	86 (77–95)	^D^0.370	0.961
ADLER score, median (IQR)	29 (28–30)	29 (21–30)	^D^0.290	0.961
Anterior elevation, median ° (IQR)	160 (134–170)	150 (115–160)	^D^0.365	0.961
Abduction, median ° (IQR)	150 (115–170)	140 (88–160)	^D^0.564	0.961
External rotation, median ° (IQR)	40 (20–49)	30 (20–45)	^D^0.676	0.961
External rotation in abduction, median ° (IQR)	75 (60–80)	70 (45–90)	^D^0.961	0.961
Internal rotation, median level reached (IQR)	L1 (L5-T12)	L4 (buttock-T12)	^D^0.380	0.961
Improvement from preoperative to final follow-up				
Subjective shoulder value (0–100), mean Δ (SD)	44.6 (24.8)	31.0 (29.0)	^B^0.197	0.961
VAS pain (0–10), mean Δ (SD)	− 4.7 (3.2)	− 4.2 (2.2)	^B^0.607	0.961
Anterior elevation, mean Δ ° (SD)	38.4 (55.9)	29.0 (52.4)	^B^0.578	0.961
External rotation, mean Δ ° (SD)	21.1 (32.1)	2.9 (27.6)	^B^0.070	0.961
Internal rotation, mean Δ* (SD)	4.7 (5.3)	− 1.4 (5.3)	^**B**^**0.00220**	**0.0352**

*BIO*-*RSA* bony increased offset reverse shoulder arthroplasty, *IQR* interquartile range, *RSA* reverse shoulder arthroplasty, *SD* standard deviation, *VAS* visual analogue scale

*Improvement measured in number of anatomic landmarks (such as one vertebra) surpassed superiorly compared with the preoperative level reached

^B^*t*-test

^D^Mann–Whitney *U* test

### Clinical outcomes

Information on clinical outcomes was available in 52 patients (74%). Postoperatively, there were no cases with an external rotation lag sign or Hornblower sign. All ROM measurements in the total cohort improved significantly compared with preoperative measurements (*p* < 0.0132), except for internal rotation (*p* = 0.052). There were no differences between the two groups in ROM at final follow-up (*p* = 1.000). The level reached in internal rotation improved by more anatomical landmarks in the BIO-RSA 38 group (Δ4.7, *SD* Δ5.3 versus Δ−1.4, *SD* Δ5.3, *p* = 0.0352, Table 5).

### Radiographic outcomes

Radiographs were available in 45 patients (59%). The interobserver reliability between the first to assessors was good for the lateralisation shoulder angle [LSA; ICC: 0.851, 95% confidence interval (CI): 0.457, 0.942] and for the inferior overhang (ICC: 0.769, 95%CI: 0.600, 0.873), and was excellent for the distalisation shoulder angle (DSA; ICC: 0.911, 95%CI: 0.842, 0.951). The reliability was poor for the radiological grading of scapular notching (*k* = 0.425), glenoid lucencies (*k*= 0.161) humeral lucencies (*k* = 0.474), ossification (*k* = 0.353) and for the assessment of graft healing (*k* = 0.068). The reliability was moderate for the assessment of graft viability (*k* = 0.644), zones of humeral lucencies (*k* = 0.581) and stress shielding (*k* = 0.536).

None of the components were considered at risk of loosening (notching grade IV, radiolucencies grade III or IV, or radiolucencies in more than three zones). Of the 25 patients with a BIO-RSA and available radiographs, the graft was considered viable in 21 cases (84%) and healed in 23 cases (92%). The inferior overhang was greater in the RSA 42 group (4.91 mm; *SD* 1.84 versus 2.96 mm; *SD* 1.80, *p* = 0.02186). The other radiographic measurements and outcomes did not significantly differ between the two groups (*p* > 0.485, Table 6).

**Table 6:** Radiographic measurements and outcomes

	BIO-RSA 38 (*n* = 25)	RSA 42 (*n* = 20)	*p*-value	Adjusted *p*-value
Lateralisation angle, mean ° (SD)	82.7 (8.2)	82.8 (8.0)	^B^0.738	1.000
Distalisation angle, mean ° (SD)	52.1 (8.1)	57.6 (9.4)	^**B**^**0.04846**	0.436
Lateralisation/distalisation, median (IQR)	1.62 (1.40–1.71)	1.42 (1.21–1.77)	^D^0.178	1.000
Inferior overhang, mean mm (SD)	2.96 (1.80)	4.91 (1.84)	^**B**^**0.002186**	**0.02186**
Notching, *n* (%)			^C^0.853	1.000
None	18 (75)	16 (80)		
Grade I	5 (21)	3 (15)		
Grade II	1 (5)	1 (4)		
Glenoid: lucency grade, *n* (%)			^C^0.708	1.000
None	23 (96)	19 (95)		
Grade I	0 (0)	1 (5)		
Grade II	1 (4)	0 (0)		
Humerus: lucencies, median *n* of zones (IQR)	0 (0–1)	0 (0–0)	^D^0.155	1.000
Humerus: highest grade of lucencies, *n* (%)			^C^0.233	1.000
None	18 (72)	18 (90)		
Grade I	4 (16)	0 (0)		
Grade II	2 (8)	2 (10)		
Grade III	1 (4)	0 (0)		
Ossification grade, *n* (%)			^C^0.492	1.000
None	18 (72)	16 (80)		
Grade I	5 (20)	3 (15)		
Grade II	2 (8)	0 (0)		
Grade III	0 (0)	1 (5)		
Stress shielding, *n* (%)	3 (12)	2 (10)	^C^1.000	1.000
Graft healed, *n* (%)	23 (92)			
Graft viable, *n* (%)	21 (84)			

*BIO*-*RSA* bony increased offset reverse shoulder arthroplasty, *mm* millimetres, *RSA* reverse shoulder arthroplasty

^A^chi-square

^B^*t*-test

^C^Fisher exact test

^D^Mann–Whitney *U* test

### Complications

Three unfavourable events occurred: one patient in the BIO-RSA 38 group suffered a periprosthetic fracture of the humeral diaphysis which healed successfully with conservative treatment. One patient in the RSA 42 group underwent a single-stage revision replacing all components 6 months after the primary RSA owing to a periprosthetic joint infection. One patient in the BIO-RSA 38 group underwent a revision owing to aseptic loosening of the glenoid 3 years after the primary RSA, in which the glenoid components were replaced and the glenoid was reconstructed with a bone graft from the iliac crest.

## Discussion

The current study aimed to compare the outcomes of RSA using a larger (size 42) glenosphere with BIO-RSA with a regular glenosphere (size 38), using a Delta Xtend prosthesis for both groups, designed as an inlay prosthesis with a 155 ° neck-shaft angle. At the final follow-up, there was no difference in postoperative ROM and PROMs between the groups. The level reached in internal rotation increased by a greater amount in the BIO-RSA 38 group (*p* = 0.0352). However, although not statistically significant, internal rotation trended towards lower preoperative values in the BIO-RSA 38 group. Furthermore, the clinical relevance of this difference is questionable. Similarly, external rotation improved markedly in the BIO-RSA group but was inferior preoperatively in this group. Both differences were not statistically significant. Apart from a greater inferior overhang in the RSA 42 group, there were no differences in radiographic measurements or outcomes. These results suggest that using a larger glenosphere size is a feasible alternative for lateralising RSA.

### Range of motion

Previous studies have found glenoid lateralisation to be associated with postoperative range of motion, alongside preoperative shoulder function, preoperative status of the rotator cuff, surgical approach and implant design [28–30]. To our knowledge, there are no previous studies directly comparing BIO-RSA with a regular glenosphere size to RSA using a larger glenosphere size. The literature comparing BIO-RSA with regular RSA, regardless of glenosphere size, is contradictory. Only a few studies report improved rotational ROM, which did not seem to translate to superior PROM results [31–33]. Similarly, literature comparing ROM between glenosphere sizes is sparse and contradictory. Some studies report superior ROM, which does not translate to superior PROM results [5–7, 34]. Our results suggest that the benefit in terms or rotational ROM when using a BIO-RSA instead of a regular RSA is matched by the benefit of using a larger glenosphere.

The increase in lateralisation when using a size 42 glenosphere, which is currently the largest commercially available glenosphere for this implant model, instead of a size 38 is minimal (2 mm) compared with the increase in lateralisation when opting for BIO-RSA (1 cm). In the current study, the poly-ethylene insert was the same size for both groups. The increased lateralisation in BIO-RSA leads to greater muscle tension, which is beneficial for movement. Despite the minimal lateralisation, using a larger glenosphere also leads to increased wrapping of the surrounding muscles around the prosthesis which also increases muscle tension. In contrast to BIO-RSA, the larger glenosphere also does not change the centre of rotation, thereby maintaining the positive effect on the deltoid moment arm that is inherent to the medialised centre of rotation in RSA. Nevertheless, increasing the size of the glenosphere also increases the dynamic anteroposterior span of the prosthesis, leading to an increased rotational arc of the humerus. This results in a more anterior position of the humerus in internal rotation, which may cause an anterior conflict between the greater tuberosity and the conjoined tendon-coracoid complex, potentially limiting internal rotation. Using BIO-RSA with a standard glenosphere does not increase the diameter of the rotational arc, potentially avoiding an anterior conflict. Further biomechanical studies are required to confirm the dynamic changes caused by increasing the glenosphere size.

Previous studies focus on objective ROM measured in clinic. However, for daily activities requiring rotational motion, more complex movements are necessary than internal or external rotation alone, such as adequate abduction and extension [35]. A previous study confirmed this discordance between objective and patient-reported range of motion [36]. To assess functional internal and external rotation in tasks of daily living the ADLIR and ADLER questionnaires were used in this study. Satisfactory results were achieved in our cohort of patients undergoing RSA and BIO-RSA (median ADLIR > 84/100 and median ADLER 29/30) and no difference was observed between the two groups.

### Radiographic parameters

Implant positioning was assessed on radiographs using the LSA, DSA, and inferior overhang. Interestingly, the angles did not differ significantly between the groups, despite inherent differences in implant positioning. A possible explanation may be the inaccuracy of these measurements on plain radiographs: the angle is highly dependent on the angle in which the radiograph is taken and the position of the arm. Furthermore, the inferior overhang was significantly lower in the BIO-RSA 38 group (*p* = 0.02186). However, the overhang is measured using lines drawn parallel to the central peg of the glenoid. In contrast to regular RSA, the glenoid component is placed in about 10 ° inferior inclination when using a BIO-RSA technique as described by Boileau et al.[37]. This results in a lower measurement than the true inferior overhang.

In the current cohort, the rate of scapular notching did not differ between the two groups (*p* = 1.000). To our knowledge, there are no previous studies comparing radiographic outcomes between BIO-RSA and regular RSA using a larger glenosphere. However, two previous studies comparing BIO-RSA with regular RSA regardless of glenosphere size found a higher rate of notching in the RSA group (75% versus 40% and 68% versus 33%, p < 0.028) [33, 38]. When a larger glenoid component is placed in the same position, more inferior overhang is created, potentially decreasing the rate of notching. One previous randomised study found a significant reduction in scapular notching rate using a larger glenoid component; 49% in patients receiving a 38-mm component, and 12% with a 42-mm component [4].

### Costs

BIO-RSA using an autograft from the humeral head is more economical compared with other lateralisation techniques, such as using an allograft or an augmented baseplate [39]. However, the added operative time and specific operative tools required for this procedure still lead to increased costs compared with regular RSA, while opting for a larger glenosphere does not increase the time or costs of the procedure. We hypothesize that regular RSA using a larger glenosphere is more cost-effective than BIO-RSA.

## Limitations

First, patients were identified retrospectively, which may lead to a selection bias owing to the factors influencing the decision to perform RSA or BIO-RSA. To address this shortcoming, patients were matched to create more comparable groups. Despite including age as a matching parameter, the age differed significantly between the groups, this may indicate that the RSA cohort was too small to achieve optimal matching. There was also a significant difference in follow-up time between the groups. This reflects current practice as BIO-RSA is becoming increasingly popular in recent years. We intentionally selected a large time window to include a large cohort, which benefits the matching accuracy. The difference in follow-up time may be a source of bias, however, a previous study found no significant changes in results after 1 year, which was the minimum follow-up in this study [8]. Furthermore, the approach differed between the groups (the anterosuperior approach was used for regular RSA and the deltopectoral approach for BIO-RSA); however, the approach did not influence outcomes in previous studies [40, 41]. Second, bone graft healing and viability, and implant positioning is best assessed on computed tomography (CT) scans instead of radiographs. However, CT scans were not available in all patients. To maintain methodological consistency, we opted to assess these factors on radiographs in all patients. Last, the current cohort is too small to compare rare complications and revisions between the two groups.

## Conclusions

At a minimum of 1 year follow-up, there was no difference in range of motion when comparing BIO-RSA with a size 38 glenosphere to RSA with a size 42 glenosphere. Similarly, no differences were found in patient-reported and radiographic results, apart from a smaller inferior overhang in the BIO-RSA group. However, prospective, randomised studies are required to confirm the findings, as well as including different prosthesis designs. Besides the similar clinical results found in this study, increasing the glenosphere size is less technically demanding and time consuming compared with BIO-RSA, less costly, and does not have technique-specific complications, such as graft non-union and resorption. These findings suggest that using a larger glenosphere size is a feasible and simple alternative to BIO-RSA for lateralising RSA. The conclusions of this study may also add perspective for manufacturers to pursue development and research towards larger (i.e. 44–46 mm) glenospheres.

### Supplementary Information

**Additional file 1: Table S1. **Radiographic analysis methods.

## Data Availability

Data will be made available upon reasonable request with the authors.
