# Supplementary material for: Lateralising reverse shoulder arthroplasty using bony increased offset (BIO-RSA) or increasing glenoid component diameter: comparison of clinical, radiographic and patient reported outcomes in a matched cohort
Source: J Orthop Traumatol. 2024 Apr 18;25:20. doi: 10.1186/s10195-024-00764-4 (PMC11026328; doi:10.1186/s10195-024-00764-4)
Supplement: Supplementary file 1 — Additional file 1: Table S1. Radiographic analysis methods. [file 10195_2024_764_MOESM1_ESM.docx]

**Supplementary Table 1: Radiographic analysis methods**

| **Angles** |  |
| --- | --- |
| Lateralisation shoulder angle | Angle measured from the superior glenoid tubercle to the most lateral border of the acromion, and from there to the most lateral border of the greater tuberosity. |
| Distalisation shoulder angle | Angle measured from the superior glenoid tubercle to the most lateral border of the acromion, and from there to the most superior border of the greater tuberosity. |
|  |  |
| **Sirveaux-Nerot classification for scapular notching** |  |
| Grade 1 | A defect which is confined to the pillar. |
| Grade 2 | A defect in contact with the lower screw. |
| Grade 3 | A defect surpassing the lower screw. |
| Grade 4 | A defect extending under the baseplate. |
|  |  |
| **Brooker classification for ossification** |  |
| Grade 1 | Islands of bone within the soft tissues around the shoulder. |
| Grade 2 | Bone spurs from the proximal humerus or scapula, leaving at least 1 centimetre between the opposing bone surfaces. |
| Grade 3 | Bone spurs from the proximal humerus or scapula, reducing the space between opposing bone surfaces to less than 1 centimetre. |
| Grade 4 | Apparent osseous ankylosis of the shoulder. |
|  |  |
| **Schoch classification for radiolucencies** |  |
| Grade 0 | No radiolucent lines |
| Grade 1 | Incomplete radiolucent lines and less than 1 millimetre wide. |
| Grade 2 | Complete radiolucent lines (spanning completely around the implant) and between 1 and 1.5 millimetres wide. |
| Grade 3 | Incomplete radiolucent lines and between 1.5 and 2 millimetres wide. |
| Grade 4 | Complete radiolucent lines and between 1.5 and 2 millimetres wide. |
| Grade 5 | Complete radiolucent lines and more than 2 millimetres wide. |
| Viability | The absence of lysis or decreased thickness of the graft |
| **For BIO-RSA:** |  |
| Healing | The absence of radiolucent lines between the bone graft and native glenoid |
| Viability | The absence of lysis or decreased thickness of the graft |
